# Investigating the impact of early-life adversity on physiological, immune, and gene expression responses to acute stress: A pilot feasibility study

**DOI:** 10.1371/journal.pone.0221310

**Published:** 2020-04-03

**Authors:** Idan Shalev, Waylon J. Hastings, Laura Etzel, Salomon Israel, Michael A. Russell, Kelsie A. Hendrick, Megan Zinobile, Sue Rutherford Siegel

**Affiliations:** 1 Department of Biobehavioral Health, The Pennsylvania State University, University Park, PA, United States of America; 2 Department of Psychology, The Hebrew University of Jerusalem, Jerusalem, Israel; 3 Scheinfeld Center of Human Genetics for the Social Sciences, Hebrew University, Jerusalem, Israel; Stellenbosch University, SOUTH AFRICA

## Abstract

**Objective:**

Exposure to early-life adversity (ELA) can result in long-term changes to physiological systems, which predispose individuals to negative health outcomes. This biological embedding of stress-responsive systems may operate via dysregulation of physiological resources in response to common stressors. The present pilot study outlines a novel experimental design to test how young adults’ exposure to ELA influences neuroendocrine and inflammatory responses to acute stress.

**Materials and methods:**

Participants were 12 males (mean age = 21.25), half of whom endorsed at least three significant adverse events up to age 18 years (‘ELA group’), and half who confirmed zero (‘controls’). Using a randomized within-subjects, between-groups experimental design, we induced acute psychosocial stress (Trier Social Stress Test, TSST), and included a no-stress control condition one week apart. During these sessions, we obtained repeated measurements of physiological reactivity, gene expression of the glucocorticoid receptor (*NR3C1*), and plasma levels of pro-inflammatory cytokines (IL-1β, IL-6, IL-8 and TNFα) over a 4-hour window post-test.

**Results:**

In this pilot study, the ELA group evinced higher cortisol response and blunted *NR3C1* gene expression in response to the TSST compared with controls, while no differences were observed in the no-stress condition. For pro-inflammatory cytokines, only IL-6 increased significantly in response to the TSST, with no differences between the two groups.

**Conclusion:**

Overall, this pilot feasibility study provides a framework to investigate the biological embedding of early-adversity via dysregulation across physiological and genomic systems in response to acute psychosocial stress. ELA may program such systems in a maladaptive manner more likely to manifest during times of duress, predisposing individuals to the negative health consequences of everyday stressors. Future studies with larger sample size including both males and females are needed to replicate and expand upon these preliminary findings.

## Introduction

An ever-growing body of research suggests that early-life adversity (ELA) can program biological systems, which predispose individuals to later-life physical and mental-health problems [1, 2]. Empirical evidence exist for associations between ELA and elevated risk of depression, cardiovascular disease, diabetes, autoimmune diseases and cancer, to name a few (reviewed in [3]). Despite the salient role of ELA on disease risk, the biological mechanisms that play a downstream role in increased disease susceptibility are not well understood.

Mechanistic research on the biological embedding of ELA has emphasized maladaptive programming of the hypothalamic-pituitary-adrenal (HPA) axis with the associate release of cortisol through processes of allostasis [3, 4]. Specifically, studies have documented a shift in HPA axis function with hyper- or hypo-secretion of cortisol in depression and post-traumatic stress disorder (PTSD), respectively [5, 6]. Similar findings have been reported in individuals exposed to ELA without such diagnoses [7, 8]. This programming, in turn, can result in mitochondrial dysfunction, failure to down-regulate the inflammatory response and overall metabolic stress, thereby increasing circulatory levels of lipids, glucose, oxidants, and pro-inflammatory cytokines [9, 10]. Further mechanistic research on the biological embedding of ELA suggests physiological dysregulation may be mediated at the genetic level via epigenetic modifications that can persist over long periods of time [11], including evidence linking ELA and cortisol responses via methylation levels in the glucocorticoid receptor (*NR3C1*) gene [12, 13]. Other research suggest the involvement of telomere biology in mediating the longer-term link between ELA and disease risk [14]. What is less clear, however, is how target immune cells respond to stress *in vivo* as a consequence of ELA, via rapid gene expression regulation [15, 16]. This new knowledge can provide insights into an integrated and dynamic cellular regulatory system whose signal profiles could forecast disease risk associated with early adversity [17–19].

Cells show remarkable flexibility in response to stimuli by regulating gene expression in a transient manner [16, 20]. In one of the first studies relating peripheral blood mononuclear cells (PBMC) gene expression to trauma, basal gene expression signatures, both immediately following trauma and four months later, distinguished survivors who met diagnostic criteria for PTSD from those who did not [21]. Follow-up studies provided further support for associations between chronic stress and basal expression levels of genes related to glucocorticoid signaling and pro- and anti-inflammatory pathways [22–24]. Importantly, several studies have also provided evidence of rapid (e.g., from 30 minutes to 8 hours) gene expression activation in response to *in vitro* stimulation [25], psychological stress [26, 27], physical stress [28] and stress-reduction methods [29]. Notably, these response patterns were recently dubbed the “conserved transcriptional response to adversity” [30]. Taken together, theory and evidence suggests that programmed immune cells of individuals exposed to early adversity may show compromised adaptation in response to acute stress, which, if repeated, may play a downstream role in disease risk.

For example, the glucocorticoid-immune signaling pathway has been implicated as a key mechanism in relation to chronic stress (i.e., caregiving, poverty [23, 31]), through reduced glucocorticoid receptor (*NR3C1*) availability, ligand binding affinity, and functional capacity to regulate gene expression. Specifically, chronic stress, via extended exposure to cortisol, is associated with reduced *NR3C1* expression, leading to glucocorticoid resistance and impaired negative feedback inhibition of the HPA axis [32]. Reduced levels of glucocorticoid receptors, in turn, bind less cortisol. This effectively decreases the number of ligand-bound receptor complexes available to translocate to the nucleus and regulate the expression of genes, including anti-inflammatory genes. Thus, reduced levels of *NR3C1* expression can lead to impaired immune function [33]. Further, evidence exists for elevated levels of pro-inflammatory cytokines in children and adults exposed to ELA [34, 35]. Dysregulation of the immune system in the context of ELA, as well as the resulting increase of pro-inflammatory cytokines, can increase risk for a host of diseases, from autoimmune to atherosclerosis and cancer [36].

Here, we delineate a program of research to study ELA-related programming of biological systems with emphasis on processes related to glucocorticoid signaling and inflammation. Experimental studies usually employ either a between- or within-person designs [37], and measure physiological or gene expression changes over relatively short time frames. Our novel program of research uses a within-person, between-groups experimental design, and aims to test whether ELA leads to dysregulation of physiological, gene expression and pro-inflammatory cytokines in response to a canonical laboratory stressor, compared with a resting control condition, and compared with individuals without exposure to ELA. The within-person control condition enables us to disentangle the effects of acute stress from noisy gene expression measurements in the same individuals. The between-person acute stress condition enables tests of potential programming of biological systems between ELA and control individuals, which theory suggests in more likely to manifest during times of duress [26, 27]. Measuring stress-induced physiological, immune, and gene expression changes enable tests across a wide array of biological and cellular pathways that may play a downstream role in disease susceptibility. This conceptual framework integrates environmental, psychological, and biological elements of what Cohen et al., termed ‘a stage model’ of stress and disease [38]. Finally, to substantiate group differences between ELA and control individuals, we compared individuals who reported at least three major traumatic events up to age 18 years [39], while the control group were selected only if they reported none.

Specifically, in this pilot feasibility study, we applied a validated screening instrument [40] to recruit 12 men, 6 of whom who endorsed at least three significant adverse events (‘ELA group’) [39], and 6 who confirmed zero (‘controls’). In a randomized within-subjects, between-groups experimental design, we induced acute stress in the lab (Trier Social Stress Test, TSST) and included a no-stress control condition separated by one week. During these sessions, we obtained repeated measurements of physiological reactivity, plasma levels of pro-inflammatory cytokines, and PBMC gene expression over a 4-hour window post-test. Primary analyses aimed to replicate previously known associations between physiological measures (i.e., blood-pressure and salivary cortisol) in response to acute laboratory stress. Our secondary analyses examined how young adults’ exposure to ELA influence neuroendocrine and inflammatory responses to acute stress compared with non-exposed individuals by testing physiological, *NR3C1* gene expression, and pro-inflammatory cytokines responses. We focused on four pro-inflammatory cytokines, including interleukin-1β (IL-1β**)**, interleukin-6 (IL-6), interleukin-8 (IL-8), and tumor necrosis factor alpha (TNF-α). Based on prior literature, we hypothesized that individuals exposed to ELA will evince dysregulated physiological, *NR3C1* gene expression, and pro-inflammatory changes to an acute laboratory stressor, a response pattern that may reveal dynamic biological signatures of early-life programming with implications for life-long health.

## Materials and methods

### Participants

Participants were healthy male college students at the Pennsylvania State University, recruited by word of mouth and advertisements on campus bulletin boards. We focused on men in this exploratory study due to known sex differences in the stress response [41] and the small sample size for stratified analyses. To obtain the sample who were exposed to ELA, a trained clinical interviewer conducted a phone interview to screen over 100 eligible men using the Stressful Life Events Screening Questionnaire (SLESQ) [40], a 13-item self-report measure that assesses lifetime exposure to traumatic events. We asked respondents 11 specific and two general categories of events, such as death of a parent or sibling, life-threatening accident, and sexual and physical abuse. Based on evidence that three or more traumatic events confers higher risk for disease [39], and considering the severity of the traumatic events, participants who responded to at least 3 incidents up to age 18 years (independently reviewed and reached consensus by MZ and IS) were invited to participate in the ELA group. Respondents’ examples for adverse exposures in this study included (unsubstantiated) child abuse and neglect, severe violence exposure, parental loss, suicide of a close friend or a family member, severe illness of an immediate family member or car accidents. In addition, the SLESQ was used to screen participants without a history of traumatic exposures to serve as the control group. Selection criteria stipulated that subjects were between 18–25 years, without current medical illness or endocrine illness (for example, asthma, diabetes, thyroid disease or pituitary gland disorders confirmed by self-report and physical examination), were currently non-smokers and were not using medication on a regular basis, including psychiatric medication. The final sample included 12 men, 6 of whom experienced early adversity (i.e., ‘ELA group’) and 6 who did not (i.e., ‘controls’) (mean age = 21.25, SD = 2.3). Demographics of the sample are presented in **Table 2**. The study was approved by the Ethics Committee at the Pennsylvania State University, registered at ClinicalTrials.gov (Identifier: NCT03637751), and all participants provided written informed consent. Participants received a modest monetary incentive for participation.

### General procedure

**Fig 1:**
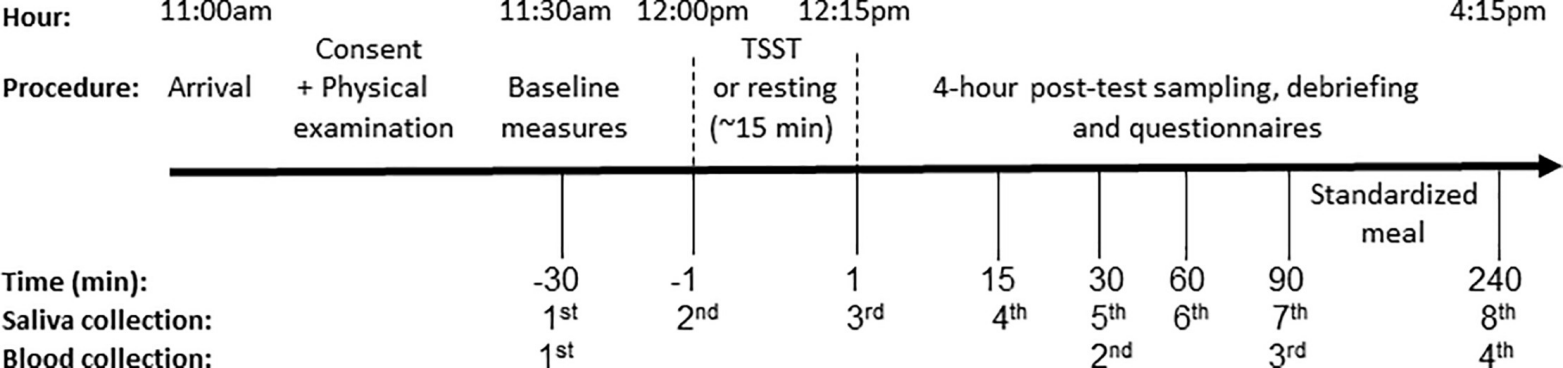
Study design for both sessions (TSST and no-stress control condition), separated by one week.

Testing was carried out at the Pennsylvania State’s Clinical Research Center (CRC). Participants made two visits to the CRC during weekdays, one week apart, on the same day. Testing was scheduled to begin at 11:00am and end by 4:15pm. We used a randomized counter-balanced order for the two sessions (i.e., TSST and no-stress control conditions) blind to participants and lab personnel. Lab personnel were also blind to group status. Participants were given specific instructions to refrain from excessive physical activity on the day of the testing, consuming alcohol for 12 hours before their arrival, and eating and drinking (besides water) for 2 hours prior to the testing session. After arrival and consent, trained nurses completed a physical examination and inserted an IV catheter into the antecubital vein 30 minutes after arrival (30 minutes prior to testing). The TSST session was scheduled to begin at 12:00pm to minimize the effects of circadian changes in cortisol, and was carried out as described previously [42]. Briefly, the TSST consists of a free speech and a mental arithmetic task of 10 minutes duration performed in front of a panel of two committee members (mixed gender) with a camera and microphone situated between the interviewers. Participants were told that they would play the role of an interviewee for a job and have 5 minutes to make an argument for their candidacy. After 5 minutes, the second task emphasizing cognitive load commenced. In this task, participants were asked to count backwards from 1,687 in multiples of 13. If a mistake was made, they were instructed to start again from the beginning. In the no-stress control condition, participants were instructed to sit in a room, read magazines, and to refrain from any stressful activities (e.g., cell-phone use was restricted). After the second blood draw, approximately 60 minutes after the TSST session and 90 minutes after the first baseline measure in the no-stress control condition, participants were administered a set of questionnaires. These questionnaires were administered in both sessions and the average score was calculated before analyses (see below for details). Considering the long time-frame of the study and the repeated collection of multiple blood samples, a standardized low-calorie meal was provided after the third blood draw (approximately at 1:45pm). Fig 1 outlines the study design.

### Primary endpoints: Physiological reactivity

Salivary cortisol was repeatedly assessed from 7 saliva samples at the following time-points: 30 minutes after arrival (30 minutes prior to testing), 1 minute prior to testing, immediately after testing (15 minutes after last sample in the control condition), and 15, 30, 60 and 90 minutes post-test. Saliva samples were kept at room temperature throughout the session, were immediately centrifuged at the end of the session at 3000 rpm at 24°C for 15 minutes, and then stored at -80°C until assayed. Systolic and diastolic blood-pressure were measured at the same time points as salivary cortisol.

Salivette swabs (Sarstedt, Germany) were used to collect saliva. Salivary cortisol was assessed, in duplicate, through an enzyme immunoassay protocol (Salimetrics) with known controls. The lower detection limit of the assay is <0.007 ug/dL. Intra-assay CV was 9.88% across all samples and inter-assay CV 5.79% across four plates. Participants’ blood pressure was measured, while seated, using an automatic monitor (Omron HEM-712C).

### Secondary endpoint: RNA extraction and gene expression assays

Gene expression changes were measured repeatedly from the four blood samples at each session at the following time-points: 30 minutes after arrival (30 minutes prior to testing), and at 30 (75 minutes after the first sample in the no-stress condition), 90 and 240 minutes post-test (Fig 1). Given known changes in immune cell redistribution and composition in response to acute stress [20], complete blood count with differential was measured within 24 hours by Quest Diagnostics using additional 4 ml EDTA collection tubes.

Whole blood samples were collected in 10 mL EDTA blood tubes via an IV catheter into the antecubital vein, and immediately centrifuged for 10 minutes at 1500g prior to collection of plasma. PBMCs were immediately isolated through density-gradient centrifugation using Ficoll. Immediately following isolation, cells were suspended in RNAlater solution (Ambion) before being stored at 4°C overnight. The duration from blood sampling to stabilization of RNA never exceeded 55 minutes. RNA extraction and cDNA synthesis were performed the following day using QIAamp RNA Blood Mini Kit and cDNA Synthesis Kit respectively (Qiagen), and then stored at -80°C until assayed. RNA purity was verified using Nanodrop 2000 spectrophotometer (Thermo Scientific).

All assays were performed on a real-time PCR (Rotor Gene Q, Qiagen). PCR reactions were set-up using the complementary QIAgility robotic pipettor (Qiagen) to ensure maximum pipetting accuracy. Samples were assayed in duplicate. All repeated, within-subject samples were run on the same plate. The reaction mix for gene expression assays consists of 5 uL TaqMan Gene Expression Master Mix (Thermo Fisher Scientific), 1x TaqMan gene expression primer, UltraPure Water (Rockland), and 100ng DNA in a 10 uL reaction. The cycling profile consists of an initial denaturing at 95°C for 15 seconds and annealing/extending at 60°C for 1 minute followed by fluorescence reading, 55 cycles. One hypothesis-driven gene (*NR3C1*: Hs00353740_m1) was normalized to a housekeeping gene (*GADD45A*: Hs00169255_m1). Expression of the *NR3C1* and the housekeeping genes were assessed on the same plate in two independent PCR reactions using cDNA from the same sample aliquot.

Sample normalization was done using the ΔΔCt method [43]. Briefly, a cycle threshold (Ct) is defined as the cycle number at which a sample’s fluorescence reaches a defined threshold. The same threshold was used for reactions assessing housekeeping and *NR3C1* genes. Thus, each sample on a given plate has two Ct values (e.g. Ct_NR3C1_ and Ct_GADD45A_). The ΔCt is calculated as the difference between the Ct of the gene of interest and the Ct of the housekeeping gene (e.g. ΔCt = Ct _NR3C1_ -Ct_GADD45A_). The ΔΔCt represents the within-subject normalization of the three post-test samples to expression levels at baseline. That is, ΔΔCt = ΔCt_POST-TEST_—ΔCt_BASELINE_. Thus, the ΔΔCt for the baseline sample for each session is always equal to zero. Lastly, fold change is calculated by exponentiating 2 by -ΔΔCt (i.e. Fold Change = 2^-ΔΔCt^). It follows that the fold change for each baseline sample is always equal to one (i.e. 2^0^).

### Secondary endpoint: Pro-inflammatory cytokines

Inflammatory assays were performed on plasma isolated from whole blood. Plasma samples were stored at -80°C prior to use. Plasma levels of IL-1β, IL-6, IL-8, and TNF-α were quantified using Meso Scale Discovery’s Multi-Array technology (MSD, V-PLEX Human Proinflammatory Panel II) and analyzed on a Meso QuickPlex SQ 120 instrument (Meso Scale Discovery, Rockville, MD, USA). Sample concentrations were determined relative to standard curves generated by fitting electrochemiluminenscent signal from stock calibrators with known concentrations using MSD Discovery Workbench® software. Samples were run in duplicate. Intra-assay variability was 8.02% across all samples and inter-assay variability was 3.87% across the three plates. The lower limits of detection for inflammatory markers were 0.646 pg/mL (IL-1β), 0.633 pg/mL (IL-6), 0.591 pg/mL (IL-8), and 0.690 pg/mL (TNF-α). Samples with concentrations below the curve fit range were assigned a value of 0 for analyses considering those analytes. This occurred for 13 samples (13.5%) for IL-1β. Samples for all other analytes were within detection ranges. Summary information for inflammatory cytokines (pg/mL) by time, session, and group status is presented in S1 Table.

### Self-reported measures and other covariates

We administered several questionnaires to assess levels of adverse exposures in the past 12 months and mental health symptoms. Specifically, participants completed the following questionnaires at both sessions; the Life-Event Stress Scale (LESS) [44], which consists of 42 common events associated with some degree of disruption of an individual's life and provide a standardized measure of the impact of a wide range of common stressors in the past year; and the Life Events Questionnaire (LEQ) [45], an 82-item inventory-type questionnaire for the measurement of life changes during the past year. The LEQ consists of items that are designed primarily for use with students. We further assessed levels of anxiety and depressive symptoms using the Beck anxiety inventory [46], Beck depression inventory [47], and State-Trait Anxiety Inventory [48], as well as perceived stress levels using the 10-item Perceived Stress Scale [49].

As noted above, given gene expression changes may depend on specific cell populations [20], we measured complete blood cell counts during both experimental sessions, as well as PBMC counts, in duplicate, using a Countess automated cell counter (Invitrogen). Other potential covariates included; age, body mass index, and socioeconomic status (i.e., parental education and income).

### Data reduction and final measures

Statistical analyses of cortisol data used log transformed cortisol values at 7 time-points and area under the curve with respect to increase (AUCi) [50]. The variables were examined for outliers (>3 SD) and none were detected. Blood pressure values were reduced to 4 measures, from 30 minutes prior to testing to 15 minutes after (samples 1–4) to evaluate the fast sympathetic response. Moreover, systolic and diastolic blood pressures were combined to derive a measure of the mean arterial pressure (MAP) to describe the average response in blood pressure (i.e., MAP = [(2 x diastolic) + systolic] / 3). Raw gene expression data was analyzed based on the 2^-(ΔΔCt) method, with normalization to a housekeeping gene, and compared to the first baseline measure in each session [43]. AUCi was computed for *NR3C1* to assess overall responses from baseline. [51]

For the four pro-inflammatory cytokines, considering high correlations [52] (Pearson correlations ranged from .30 to .72), principal component analysis (PCA) indexing *systemic inflammation* of IL-1β, IL-6, IL-8 and TNF-α measures was conducted for the four repeated measures using data from both sessions. In each instance, the first component was extracted for use in subsequent analyses. The four repeated items mapped to components with eigenvalues of 2.55 for the first time point, which explained 63.81% of the variance across all four cytokines, 2.29 for the second time point (57.25% of variance), 2.46 for the third time point (61.54% of variance), and 2.80 for the fourth time point (69.88% of variance). PCA of AUCi for all four cytokines yielded two components with eigenvalues 1.93 and 1.01, which explained 48.27% and 25.37% of the variance respectively. Closer inspection of the factor loading scores for the PCA of AUCi revealed that the first component was largely representative of three cytokines (IL1-β, IL-8, TNF-α) with the second representing IL-6 (Table 1). PCA was also conducted on the four repeated measures independently within each session (TSST and no-stress). The four repeated items in the TSST session mapped onto components with eigenvalues 2.02–2.56, which explained 50.41%-64.01% of variance at each time point. The four repeated items in the no-stress session mapped onto components with eigenvalues 1.65–2.29, which explained 41.28%-57.16% of variance at each time point. The components mapped using data from both sessions were used to investigate within-person differences across sessions, while the components mapped within each session independently were used to investigate between-person differences (i.e. ELA status) within each session. Scores in all repeated questionnaires for both sessions were averaged to increase reliability (Pearson correlations ranged from .72 to .93). None of the demographics measures differed significantly between the ELA and control groups (Table 2), and thus were not included as covariates in the analysis.

**Table 1:** PCA of pro-inflammatory cytokine AUCi: Factor loading scores.

Cytokine	Factor 1	Factor 2
IL1-β	0.597	0.224
IL-6	0.174	0.942
IL-8	0.908	-0.080
TNF-α	0.849	-0.265

**Table 2:** Sample characteristics.

Variable, mean (SD)	Total (N = 12)	Control (N = 6)	ELA (N = 6)	P value diff
Age	21.25 (2.3)	20.83 (1.6)	21.67 (2.9)	0.56
SES (average)	2.83	2.83	2.83	0.23^1^
1. Working class	1	0	1	
2. Lower middle	2	1	1	
3. Middle	7	5	2	
4. Upper middle	2	0	2	
BMI	25.40 (3.7)	26.26 (3.7)	24.55 (4.0)	0.46
BAI	8.83 (8.2)	6.00 (5.8)	11.67 (9.8)	0.25
BDI	5.79 (6.3)	3.00 (1.3)	8.58 (8.2)	0.16
STAI	70.38 (20.4)	61.58 (9.0)	79.17 (25.5)	0.14
LESS	156.50 (84.9)	114.92 (60.9)	198.08 (89.4)	0.09
LEQ	20.21 (12.5)	13.75 (5.0)	26.67 (14.8)	0.07
PSS- TSST	16.08 (8.6)	11.00 (5.3)	21.67 (8.6)	0.03
PSS- no-stress	15.75 (10.0)	9.50 (4.9)	22.00 (10.1)	0.02

^1^p-value from Chi-square

SES- socioeconomic status; BMI- body mass index; BAI- Beck anxiety inventory; BDI- Beck depression inventory; STAI- state-trait anxiety inventory; LESS- life-event stress scale; LEQ- life events questionnaire; PSS- perceived stress scale.

### Statistical analysis

All statistical tests were carried out using SPSS version 25 (Windows). Repeated measures general linear models (GLMs), ordinary least squares multiple regression analyses, Pearson product-moment correlations, and t-tests were carried out as appropriate. Statistical analyses of changes in gene expression, physiological responses and cytokines levels were subjected to multivariate GLMs, with salivary cortisol, cytokines and gene expression as the repeated measure, condition (stress/no stress) as a within-subjects factor, and status (risk/control) as between-subjects factors. In addition to these analyses, univariate tests were applied to summary cortisol measures (AUCi, [50]) to ascertain reliability of findings, as well as blood pressure (MAP), gene expression, and pro-inflammatory cytokine measures. Huynh-Feldt corrections were applied if sphericity (significant differences in variance between groups) was significant, and only adjusted results are reported.

Adjustment for multiple comparisons was performed using Bonferroni correction in accordance with FDA guidelines for multiplicity in clinical trials [53]. As the primary endpoints, analyses for MAP and cortisol were controlled for collectively, and analyses within each secondary endpoint (i.e. gene expression, cytokine PCA, and raw cytokines) were adjusted independently. For the purposes of multiplicity, analyses within subgroups defined by ELA status were each considered additional tests to be accounted for. Results following adjustment and domains within which analyses were adjusted are summarized in S2 Table.

## Results

### Sample characteristics and self-reported measures

The ELA and control group did not differ in demographics measures (i.e., age, socioeconomic status and body max index) (Table 2). While group differences existed in exposure to traumatic events up to age 18 (i.e. SLESQ≥3 for ELA; SLESQ = 0 for controls), the ELA and control groups did not differ significantly in their experiences of stressful life events during the past year (univariate ANOVA between-subjects effect: F = 3.55, p = 0.089 for LESS, estimated effect size η^2^ = 0.26; F = 4.10, p = 0.070 for LEQ, estimated effect size η^2^ = 0.29). There were no significant differences in levels of anxiety [48] (F = 2.54, p = 0.142, estimated effect size η^2^ = 0.20) or depressive symptoms [47] (F = 2.73, p = 0.129, estimated effect size η^2^ = 0.22) between the two groups, although levels tended to be higher in the ELA group relative to controls for all measures of stress, anxiety, and depression (Table 2). Further, the ELA group self-reported significantly greater perceived stress in the TSST session compared with controls (F = 6.07, p = 0.033, effect size η^2^ = 0.38), as well as in the no-stress condition (F = 7.49, p = 0.021, effect size η^2^ = 0.43), and tended to report more stress in response to the TSST (Likert scale from 1–10) (F = 4.02, p = 0.080).

### Physiological, gene expression, and pro-inflammatory cytokines response to acute psychosocial stress compared with a no-stress control condition

#### Primary endpoints: Physiological

**Fig 2:**
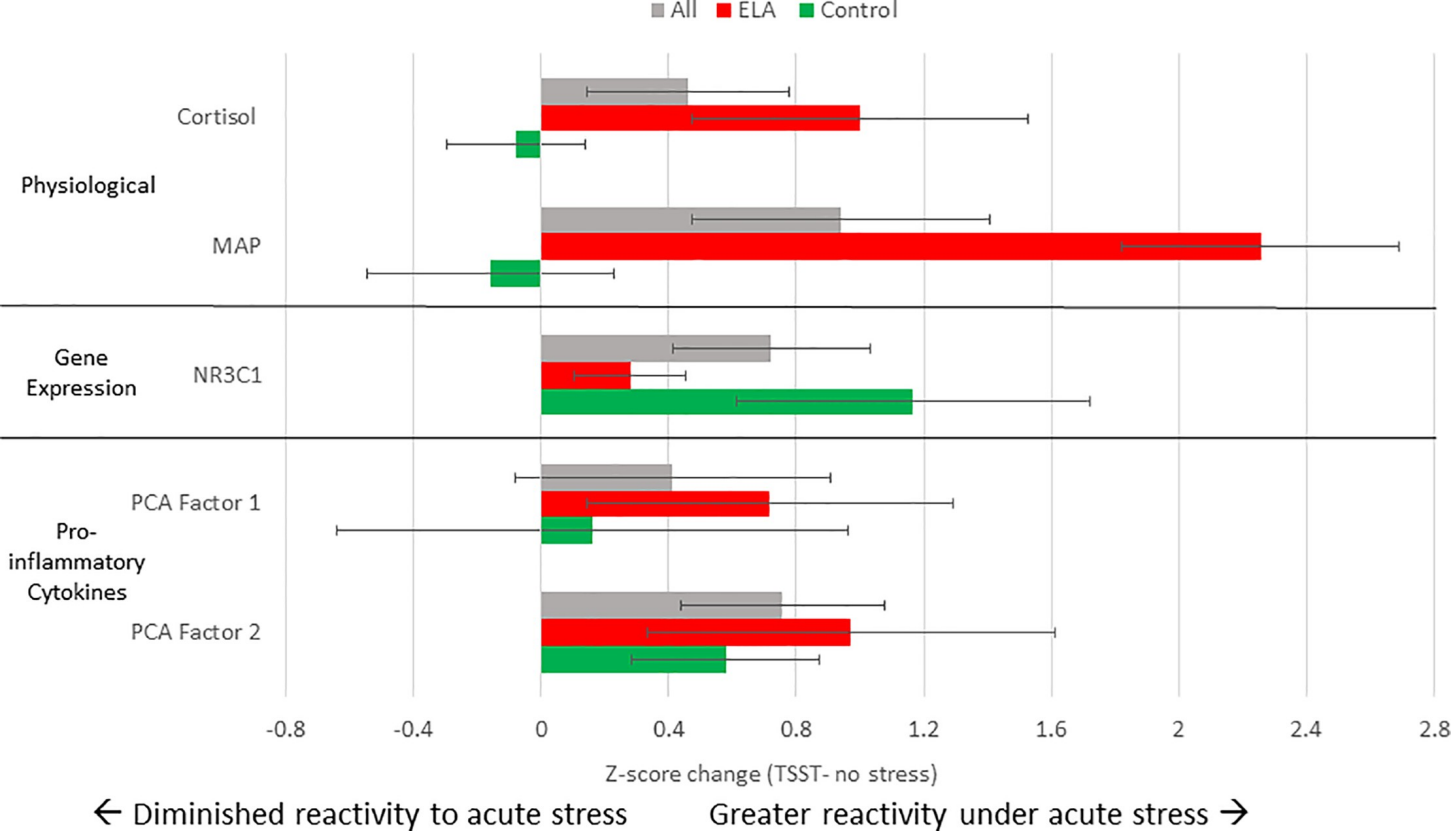
Normalized change score for physiological, gene expression, and pro-inflammatory cytokine response to the TSST relative to the no-stress session for ELA group, control group and full sample. Change scores were calculated by standardizing summary AUCi using data from both sessions and subtracting participant values from the no-stress session from those in the TSST session. Error bars represent standard error of the mean. Change scores are expressed for the full sample (grey), ELA group (red), and control group (green).

In the whole sample, repeated measures GLMs indicated significant within-subjects effect for salivary cortisol in response to the TSST, compared with a no-stress condition (Time x Session, F = 4.47, p = 0.003, estimated effect size η^2^ = 0.17), as well as for mean arterial pressure (Time x Session, F = 5.31, p = 0.003, η^2^ = 0.20). Compared with controls, the ELA group exhibited significantly higher mean arterial pressure response to the TSST (Time x Status, F = 8.59, p<0.001, η^2^ = 0.46), and a non-significant trend towards a higher cortisol response in the TSST relative to no-stress (ΔAUCi: F = 3.58, p = 0.088) (Fig 2). Notably, no significant differences were observed between the ELA and control groups in the no-stress condition (Time x Status, F = 1.38, p = 0.257 for salivary cortisol; F = 1.01, p = 0.402 for MAP). Overall, these findings confirm some [54], but not all studies [7], indicating increased physiological reactivity to acute stress in young adults exposed to early adversity, compared with non-exposed individuals.

#### Secondary endpoint: Gene expression

**Fig 3:**
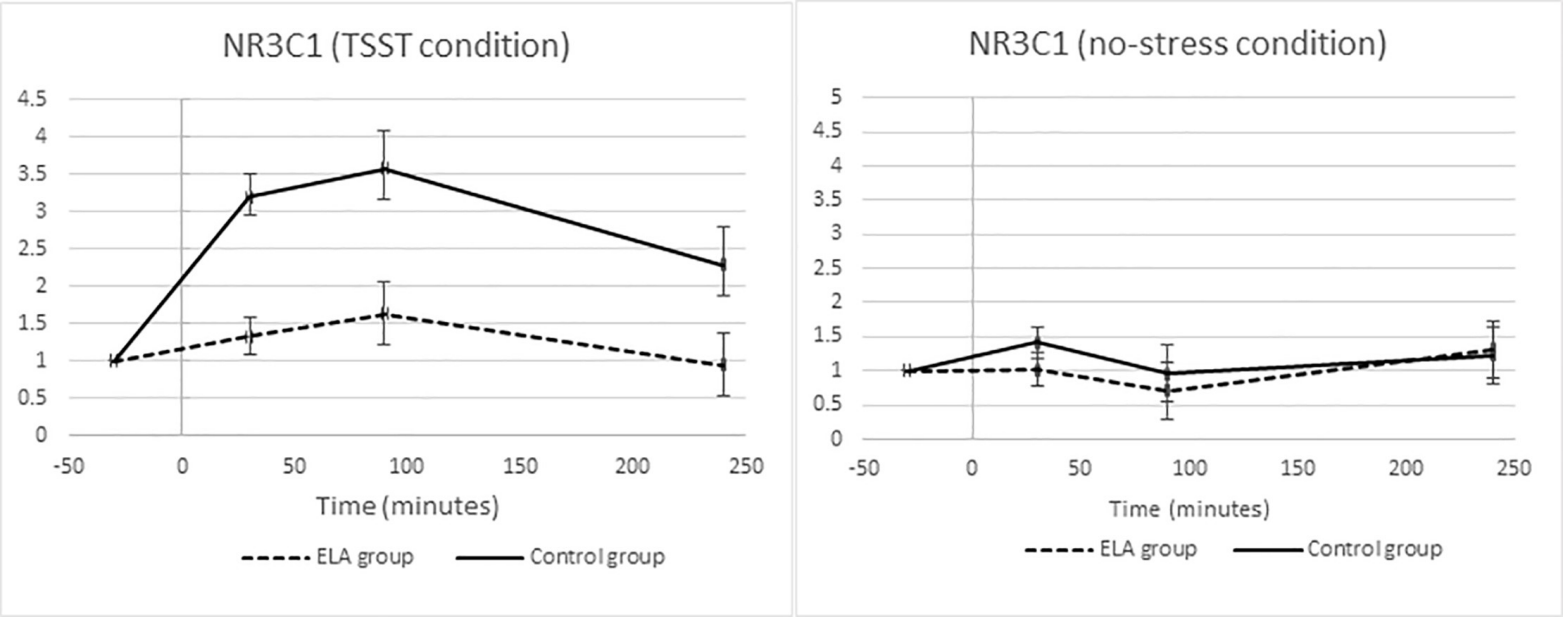
**Fold change in NR3C1 for ELA (dashed lines) and control groups (solid lines) in response to the TSST (left) and during the no-stress sessions (right).** Error bars represent standard error of the mean.

In the whole sample, there was a significant within-subjects effect of TSST vs. no-stress condition on *NR3C1* gene expression with increased levels in the TSST (Time x Session, F = 4.85, p = 0.006, estimated effect size η^2^ = 0.19). Group analysis revealed increased levels in the control group (Time x Session, F = 5.09, p = 0.013, estimated effect size η^2^ = 0.36), but not in the ELA group, which had a blunted response to the TSST (Time x Session, F = 1.00, p = 0.406, estimated effect size η^2^ = 0.09) (Fig 3A), suggestive of *NR3C1* expression resistance and lower levels to inhibit the HPA axis. Notably, no differences were observed in *NR3C1* expression between the ELA and control groups in the no-stress condition (Time x Session, F = 0.78, p = 0.491) (Fig 3B).

#### Secondary endpoint: Pro-inflammatory cytokines

In the whole sample, PCA for the four repeated measures of IL-1β, IL-6, IL-8 and TNF-α did not reveal a significant within-subjects effect of TSST vs. no-stress condition using repeated measures GLM analysis (Time x Session, F = 0.29, p = 0.831). Similarly, an analysis of the first AUCi component did not reveal significant differences in systemic inflammation between the TSST and no-stress conditions (F = 2.09, p = 0.165). However, an analysis of the second AUCi component (largely representing IL-6) showed significantly greater pro-inflammatory responses to the TSST relative to the no-stress condition (F = 5.85, p = 0.026) (Fig 2). No differences were observed between the ELA and control groups in response to the TSST relative to no-stress using either AUCi PCA components.

Exploratory analyses of each pro-inflammatory cytokine revealed a significant within-subjects effect for IL-6 in response to the TSST (Time x Session, F = 2.97, p = 0.044, AUCi: F = 7.70, p = 0.018), but not for the other three cytokines (IL-1β, Time x Session, F = 0.80, p = 0.500, AUCi, F = 1.37, p = 0.257; IL-8, Time x Session, F = 0.85, p = 0.470, AUCi, F = 0.93, p = 0.434; TNF-α, Time x Session, F = 0.25, p = 0.863, AUCi, F = 0.40, p = 0.537).

### Adjustment for multiple comparisons & sensitivity analyses

Bonferroni correction for multiple comparisons was performed across analyses of primary endpoints and within analyses of each secondary endpoint in accordance with FDA guidelines for multiplicity in clinical trials [53]. All significant findings within primary endpoints passed correction for multiple testing at a false discovery rate of 0.05, as did observed differences in *NR3C1* gene expression between sessions and between ELA status groups within the stress session. By contrast, results of analyses with inflammatory principal components, as well as those with raw inflammatory cytokines, did not pass correction for multiple testing. A summary of results and Bonferroni-corrected p-values is provided in S2 Table.

Sensitivity analyses were conducted using the ‘leave-one-out’ method. Overall, results were robust to the removal of any individual participant. Differences in sample characteristics and self-report measures remained consistent upon removal of any given participant, as did physiological, inflammatory, and gene expression responses to the TSST relative to the no-stress session. Likewise, the ELA group continued to display increased MAP responses to the TSST relative to the control group. Differences between ELA and control groups in cortisol response to the TSST relative to no-stress (ΔAUCi) were modestly attenuated by removal of any given participant, but not appear to be driven by a single individual. S3–S5 Tables provide the full results from ‘leave one out’ sensitivity analyses.

## Discussion

This pilot study delineates a program of research to study ELA-related programming of biological systems using a within-person, between-groups experimental design. To our knowledge, this is the first investigation of stress-induced gene expression and pro-inflammatory cytokines changes within-individuals, comparing stratified groups of ELA-exposed and control individuals. By comparing a validated laboratory-based stressor to a no-stress condition within the same individuals, we are able to disentangle the effects of acute stress from noisy measurements in the same individuals. Further, this design allow us to distinctly identify if/when differences between ELA-exposed and control individuals are context dependent (i.e. manifesting only during stress). Preliminary results provide evidence in humans of a dysregulated pattern of *NR3C1* gene expression activation as a consequence of ELA. Importantly, these changes manifest more acutely in the presence of stress-induced cortisol release as compared to a no-stress resting condition.

As predicted by previous research, the ELA group evince higher cortisol response and lower *NR3C1* gene expression in response to the TSST compared with controls, with no difference between groups in the no-stress condition. For pro-inflammatory cytokines, only IL-6 increased significantly in response to the laboratory-induced stressor. Overall, this pilot study provides a framework to investigate the biological embedding of ELA via a dynamic and dysregulated pattern spanning multiple levels of analysis (genomic and physiological), and affords the opportunity to parse out whether such dysregulation is context dependent (i.e. manifesting only during duress). This conceptual framework integrates multiple definitions of stress and disease at the environmental (e.g., ELA), psychological (e.g., psychosocial stress response), and biological levels (e.g., physiological, gene expression and pro-inflammatory cytokine responses) [38].

These preliminary findings concur with the receptor-mediated model of glucocorticoid signaling resistance [55]. First, ELA was associated with increased physiological response to the TSST compared with controls, confirming some [54], but not all studies [7], indicating increased physiological reactivity in young adults exposed to early adversity. Second, chronic exposure to cortisol, as a consequence of ELA, can lead to a compensatory response whereby glucocorticoid sensitivity decreases (e.g. via decreased receptor availability). Here we replicated prior evidence of reduced *NR3C1* expression levels in ELA-exposed individuals, but only in response to acute laboratory stress. Third, in vitro studies have established a connection between glucocorticoid exposure and diminished capacity of immune cells to inhibit pro-inflammatory cytokines in individuals exposed to psychological stress. Here, only pro-inflammatory cytokine IL-6 increased significantly in response to acute stress, replicating previous studies [56].

Methodological strengths of this program of research include a laboratory-based within-subjects experimental design, which allows stronger causal inferences. We collected repeated measurements over a relatively long time scale to document changes in gene expression and pro-inflammatory cytokines. Our within-subjects, between-groups design, combined with four repeated measurements in each session, reduced biological variability and increased power to detect true associations. Although this preliminary study lacked sufficient power to investigate three-way interactions (i.e. Time x Session x Status) or perform multivariate analyses across outcomes, the strength of the design affords the opportunity to test for such associations at comparably smaller sample sizes than studies without as many repeated measures. Furthermore, inclusion of a no-stress control session allows researchers the chance to identify differences that are context (i.e. stress) dependent.

We acknowledge limitations. First, this was a pilot feasibility study with a small sample size. Although comparable to similar prior investigations [26, 31, 57], the results from this study still need to be interpreted with caution. Notwithstanding, the strength of the within-subjects experimental design combined with the leave-one-out sensitivity analysis and adjustment for multiple comparisons alleviate concerns about spurious findings of the results reported herein. Second, we focused on men in this exploratory study due to known sex differences in the stress response [41] and the small sample size for stratified analyses. Future studies with larger sample size including both males and females are needed to replicate these findings. Third, we focused on a single hypothesis-driven gene. There are multiple biological pathways that are activated in response to stress that may play a downstream role in disease susceptibility, such as the conserved transcriptional response to adversity pathway [30]. Prior research has investigated multiple genes using microarrays [23, 26–28, 31]. Future studies with adequate sample size will benefit by testing larger groups of genes/pathways. Fourth, this study did not consider specific types of ELA, or timing of exposure. Here, we focused specifically on severity of multiple (i.e., minimum of three) ELA exposures up to age 18 years. Future research can explore specific types of ELA in different populations and settings. Fifth, while this controlled laboratory experimental design allows stronger causal inferences, there are other alternative explanatory variables that can explain individual differences in response to stress (e.g., temperament), which can be considered in future studies. Further, our study included non-Hispanic white males and thus future research need to test whether the association generalizes to other populations. Finally, although we included a no-stress condition to control for the higher degree of noise associated with gene expression measurements, the control session did include the stress of venipuncture. However, this is unavoidable technical limitation for collecting sufficient immune cells of high quality for gene expression research.

In conclusion, ELA may program physiological systems in a maladaptive manner more likely to manifest during times of duress, predisposing individuals to the negative health consequences of everyday stressors. Although increased activation of the glucocorticoid-immune signaling in response to acute stress is considered adaptive in the short-term, persistent activation can increase risk for mental and physical health problems. This program of research and preliminary analyses provides a framework to investigate new targets for therapeutic interventions mitigating the negative effects of early adversity, such as pharmacological agents acting on the glucocorticoid receptor [58]. Further, while previous risk factors and biomarkers of stress contributed to our understanding of biological embedding processes, these are nevertheless static characteristics that have not explained health outcomes very well. For example, considering high failure rates for depression treatments, and in order to tailor individual interventions, identifying objective changes in stress-induced gene expression may help to predict short-term intervention efficacy in clinical and non-clinical settings. An example for such an effort could be to utilize models of dynamic cellular markers as individual-level factors to account for variation in intervention response and clinical outcomes [17–19]. Thus, future research in this area can have a range of impacts for basic science, intervention studies and clinical practice that will influence treatments to match the specific cellular processes operating within an individual.

## Supporting information

S1 Table Summary information for inflammatory cytokines (pg/mL) by time, session, and group status. Data presented as sMean (Standard Deviation).
(DOCX)

S2 Table False discovery rate adjustment across study primary and secondary endpoints. Adjustment was performed across both primary outcomes (cortisol and MAP) and within each secondary outcome. Significant results are bolded. *Univariate analyses of raw cytokine AUCi were considered confirmatory following results of repeated measures models, and thus were not included in the count of total tests within raw cytokines secondary endpoint.
(DOCX)

S3 Table Leave one out sensitivity analyses of Sample Characteristics (p-value). Original results shown at top. Results which vary in significance from main findings are bolded.
(DOCX)

S4 Table Leave one out sensitivity analyses of stress-induced changes in physiological, gene expression, and pro-inflammatory cytokines (p-value). Original results shown at top. Results which vary in significance from main findings are bolded.
(DOCX)

S5 Table Leave one out sensitivity analyses of group differences in stress-induced changes in physiological, gene expression, and pro-inflammatory cytokines (p-value). Original results shown at top. Results which vary in significance from main findings are bolded.
(DOCX)

## References

[1] Widom C.S., et al, A prospective investigation of physical health outcomes in abused and neglected children: New findings from a 30-year follow-up. American journal of public health, 2012 102(6): p. 1135–1144. 10.2105/AJPH.2011.300636 22515854 PMC3483964

[2] Ben-Shlomo Y. and Kuh D., A life course approach to chronic disease epidemiology: conceptual models, empirical challenges and interdisciplinary perspectives. 2002, Oxford University Press. 11980781

[3] Cohen S., Janicki-Deverts D., and Miller G.E., Psychological stress and disease. JAMA, 2007 298(14): p. 1685–7. 10.1001/jama.298.14.1685 17925521

[4] Danese A. and McEwen B.S., Adverse childhood experiences, allostasis, allostatic load, and age-related disease. Physiology & behavior, 2012 106(1): p. 29–39. 21888923 10.1016/j.physbeh.2011.08.019

[5] Meewisse M.-L., et al, Cortisol and post-traumatic stress disorder in adults: systematic review and meta-analysis. The British Journal of Psychiatry, 2007 191(5): p. 387–392. 17978317 10.1192/bjp.bp.106.024877

[6] Burke H.M., et al, Depression and cortisol responses to psychological stress: a meta-analysis. Psychoneuroendocrinology, 2005 30(9): p. 846–856. 10.1016/j.psyneuen.2005.02.010 15961250

[7] Bunea I.M., Szentágotai-Tătar A., and Miu A.C., Early-life adversity and cortisol response to social stress: a meta-analysis. Translational psychiatry, 2017 7(12): p. 1274 10.1038/s41398-017-0032-3 29225338 PMC5802499

[8] Hunter A.L., Minnis H., and Wilson P., Altered stress responses in children exposed to early adversity: a systematic review of salivary cortisol studies. Stress, 2011 14(6): p. 614–626. 10.3109/10253890.2011.577848 21675865

[9] Miller G.E., Chen E., and Parker K.J., Psychological stress in childhood and susceptibility to the chronic diseases of aging: moving toward a model of behavioral and biological mechanisms. Psychological bulletin, 2011 137(6): p. 959 10.1037/a0024768 21787044 PMC3202072

[10] Picard M., Juster R.P., and McEwen B.S., Mitochondrial allostatic load puts the 'gluc' back in glucocorticoids. Nat Rev Endocrinol, 2014 10(5): p. 303–10. 10.1038/nrendo.2014.22 24663223

[11] Anacker C., O'Donnell K.J., and Meaney M.J., Early life adversity and the epigenetic programming of hypothalamic-pituitary-adrenal function. Dialogues in clinical neuroscience, 2014 16(3): p. 321 25364283 10.31887/DCNS.2014.16.3/canacker PMC4214175

[12] Alexander N., et al, Glucocorticoid receptor gene methylation moderates the association of childhood trauma and cortisol stress reactivity. Psychoneuroendocrinology, 2018 **90**: p. 68–75. 10.1016/j.psyneuen.2018.01.020 29433075

[13] Edelman S., et al, Epigenetic and genetic factors predict women's salivary cortisol following a threat to the social self. PLoS One, 2012 7(11): p. e48597 10.1371/journal.pone.0048597 23155396 PMC3498240

[14] Shalev I., Early life stress and telomere length: investigating the connection and possible mechanisms: a critical survey of the evidence base, research methodology and basic biology. Bioessays, 2012 34(11): p. 943–52. 10.1002/bies.201200084 22991129 PMC3557830

[15] Lopez-Maury L., Marguerat S., and Bahler J., Tuning gene expression to changing environments: from rapid responses to evolutionary adaptation. Nat Rev Genet, 2008 9(8): p. 583–93. 10.1038/nrg2398 18591982

[16] de Nadal E., Ammerer G., and Posas F., Controlling gene expression in response to stress. Nat Rev Genet, 2011 12(12): p. 833–45. 10.1038/nrg3055 22048664

[17] Antoni M.H., et al, Cognitive-behavioral stress management reverses anxiety-related leukocyte transcriptional dynamics. Biological psychiatry, 2012 71(4): p. 366–372. 10.1016/j.biopsych.2011.10.007 22088795 PMC3264698

[18] West M., et al, Predicting the clinical status of human breast cancer by using gene expression profiles. Proceedings of the National Academy of Sciences of the United States of America, 2001 98(20): p. 11462–11467. 10.1073/pnas.201162998 11562467 PMC58752

[19] van't Veer L.J., et al, Gene expression profiling predicts clinical outcome of breast cancer. Nature, 2002 415(6871): p. 530–536. 10.1038/415530a 11823860

[20] Cole S.W., Elevating the perspective on human stress genomics. Psychoneuroendocrinology, 2010 35(7): p. 955–962. 10.1016/j.psyneuen.2010.06.008 20630660 PMC2917592

[21] Segman R.H., et al, Peripheral blood mononuclear cell gene expression profiles identify emergent post-traumatic stress disorder among trauma survivors. Mol Psychiatry, 2005 10(5): p. 500–13, 425. 10.1038/sj.mp.4001636 15685253

[22] Miller G., Rohleder N., and Cole S.W., Chronic interpersonal stress predicts activation of pro-and anti-inflammatory signaling pathways six months later. Psychosomatic medicine, 2009 71(1): p. 57 10.1097/PSY.0b013e318190d7de 19073750 PMC2720615

[23] Miller G.E., et al, Low early-life social class leaves a biological residue manifested by decreased glucocorticoid and increased proinflammatory signaling. Proceedings of the National Academy of Sciences, 2009 106(34): p. 14716–14721. 10.1073/pnas.0902971106 PMC2732821 19617551

[24] Cole S.W., et al, Social regulation of gene expression in human leukocytes. Genome biology, 2007 8(9): p. R189 10.1186/gb-2007-8-9-r189 17854483 PMC2375027

[25] Moriconi F., et al, Quantitative gene expression of cytokines in peripheral blood leukocytes stimulated in vitro: modulation by the anti-tumor nerosis factor-alpha antibody infliximab and comparison with the mucosal cytokine expression in patients with ulcerative colitis. Translational Research, 2007 150(4): p. 223–232. 10.1016/j.trsl.2007.04.004 17900510

[26] Nater U.M., et al, Impact of acute psychosocial stress on peripheral blood gene expression pathways in healthy men. Biological psychology, 2009 82(2): p. 125–132. 10.1016/j.biopsycho.2009.06.009 19577611 PMC7116965

[27] Schwaiger M., et al, Altered stress-induced regulation of genes in monocytes in adults with a history of childhood adversity. Neuropsychopharmacology, 2016 41(10): p. 2530 10.1038/npp.2016.57 27091381 PMC4987852

[28] Carlson L.A., et al, Changes in transcriptional output of human peripheral blood mononuclear cells following resistance exercise. European journal of applied physiology, 2011 111(12): p. 2919–2929. 10.1007/s00421-011-1923-2 21437602 PMC4358769

[29] Kaliman P., et al, Rapid changes in histone deacetylases and inflammatory gene expression in expert meditators. Psychoneuroendocrinology, 2014 40: p. 96–107. 10.1016/j.psyneuen.2013.11.004 24485481 PMC4039194

[30] Cole S.W., Human social genomics. PLoS genetics, 2014 10(8): p. e1004601 10.1371/journal.pgen.1004601 25166010 PMC4148225

[31] Miller G.E., et al, A functional genomic fingerprint of chronic stress in humans: blunted glucocorticoid and increased NF-κB signaling. Biological psychiatry, 2008 64(4): p. 266–272. 10.1016/j.biopsych.2008.03.017 18440494 PMC2581622

[32] Silverman M.N. and Sternberg E.M., Glucocorticoid regulation of inflammation and its functional correlates: from HPA axis to glucocorticoid receptor dysfunction. Annals of the New York Academy of Sciences, 2012 1261(1): p. 55–63. 22823394 10.1111/j.1749-6632.2012.06633.x PMC3572859

[33] Beck I.M., et al, Crosstalk in inflammation: the interplay of glucocorticoid receptor-based mechanisms and kinases and phosphatases. Endocrine reviews, 2009 30(7): p. 830–882. 10.1210/er.2009-0013 19890091 PMC2818158

[34] Danese A., et al, Childhood maltreatment predicts adult inflammation in a life-course study. Proc Natl Acad Sci U S A, 2007 104(4): p. 1319–24. 10.1073/pnas.0610362104 17229839 PMC1783123

[35] Danese A., et al, Biological embedding of stress through inflammation processes in childhood. Molecular psychiatry, 2011 16(3): p. 244 10.1038/mp.2010.5 20157309 PMC4212809

[36] Elenkov I.J. and Chrousos G.P., Stress hormones, Th1/Th2 patterns, pro/anti-inflammatory cytokines and susceptibility to disease. Trends in Endocrinology & Metabolism, 1999 10(9): p. 359–368. 10511695 10.1016/s1043-2760(99)00188-5

[37] Keren G., Between-or within-subjects design: A methodological dilemma. A Handbook for Data Analysis in the Behaviorial Sciences, 2014 **1**: p. 257–272.

[38] Cohen S., Gianaros P.J., and Manuck S.B., A stage model of stress and disease. Perspectives on Psychological Science, 2016 11(4): p. 456–463. 10.1177/1745691616646305 27474134 PMC5647867

[39] Felitti V.J., et al, Relationship of childhood abuse and household dysfunction to many of the leading causes of death in adults: The Adverse Childhood Experiences (ACE) Study. American journal of preventive medicine, 1998 14(4): p. 245–258. 10.1016/s0749-3797(98)00017-8 9635069

[40] Goodman L.A., et al, Assessing traumatic event exposure: general issues and preliminary findings for the Stressful Life Events Screening Questionnaire. J Trauma Stress, 1998 11(3): p. 521–42. 10.1023/A:1024456713321 9690191

[41] Kirschbaum C., et al, Impact of gender, menstrual cycle phase, and oral contraceptives on the activity of the hypothalamus-pituitary-adrenal axis. Psychosomatic medicine, 1999 61(2): p. 154–162. 10.1097/00006842-199903000-00006 10204967

[42] Shalev I., et al, BDNF Val66Met polymorphism is associated with HPA axis reactivity to psychological stress characterized by genotype and gender interactions. Psychoneuroendocrinology, 2009 34(3): p. 382–388. 10.1016/j.psyneuen.2008.09.017 18990498

[43] Livak K.J. and Schmittgen T.D., Analysis of relative gene expression data using real-time quantitative PCR and the 2(T)(-Delta Delta C) method. Methods, 2001 25(4): p. 402–408. 10.1006/meth.2001.1262 11846609

[44] Holmes T.H. and Rahe R.H., The Social Readjustment Rating Scale. J Psychosom Res, 1967 11(2): p. 213–8. 10.1016/0022-3999(67)90010-4 6059863

[45] Sarason I.G., Johnson J.H., and Siegel J.M., Assessing the impact of life changes: development of the Life Experiences Survey. Journal of consulting and clinical psychology, 1978 46(5): p. 932 10.1037//0022-006x.46.5.932 701572

[46] Beck A.T., et al, An inventory for measuring clinical anxiety: psychometric properties. J Consult Clin Psychol, 1988 56(6): p. 893–7. 10.1037//0022-006x.56.6.893 3204199

[47] Beck A.T., et al, Comparison of Beck Depression Inventories -IA and -II in psychiatric outpatients. J Pers Assess, 1996 67(3): p. 588–97. 10.1207/s15327752jpa6703_13 8991972

[48] Spielberger C.D., Gorsuch R.L., and Lushene R.E., Manual for the state-trait anxiety inventory. 1970.

[49] Cohen S., Kamarck T., and Mermelstein R., A global measure of perceived stress. J Health Soc Behav, 1983 24(4): p. 385–96. 6668417

[50] Pruessner J.C., et al, Two formulas for computation of the area under the curve represent measures of total hormone concentration versus time-dependent change. Psychoneuroendocrinology, 2003 28(7): p. 916–31. 10.1016/s0306-4530(02)00108-7 12892658

[51] Miller R., et al, How to disentangle psychobiological stress reactivity and recovery: A comparison of model-based and non-compartmental analyses of cortisol concentrations. Psychoneuroendocrinology, 2018 90: p. 194–210. 10.1016/j.psyneuen.2017.12.019 29370954

[52] Muylle L., et al, Increased tumor necrosis factor alpha (TNF alpha), interleukin 1, and interleukin 6 (IL‐6) levels in the plasma of stored platelet concentrates: relationship between TNF alpha and IL‐6 levels and febrile transfusion reactions. Transfusion, 1993 33(3): p. 195–199. 10.1046/j.1537-2995.1993.33393174443.x 8438219

[53] Food and D. Administration, Multiple endpoints in clinical trials: guidance for industry. College Park, Maryland: Food and Drug Administration, 2017.

[54] Miller G.E., Chen E., and Zhou E.S., If it goes up, must it come down? Chronic stress and the hypothalamic-pituitary-adrenocortical axis in humans. Psychological bulletin, 2007 133(1): p. 25 10.1037/0033-2909.133.1.25 17201569

[55] Miller G.E., Cohen S., and Ritchey A.K., Chronic psychological stress and the regulation of pro-inflammatory cytokines: a glucocorticoid-resistance model. Health psychology, 2002 21(6): p. 531 10.1037//0278-6133.21.6.531 12433005

[56] Steptoe A., Hamer M., and Chida Y., The effects of acute psychological stress on circulating inflammatory factors in humans: a review and meta-analysis. Brain, behavior, and immunity, 2007 21(7): p. 901–912. 10.1016/j.bbi.2007.03.011 17475444

[57] Rohleder N., et al, No response of plasma substance P, but delayed increase of interleukin-1 receptor antagonist to acute psychosocial stress. Life Sciences, 2006 78(26): p. 3082–3089. 10.1016/j.lfs.2005.12.016 16414081

[58] Binder E.B., The role of FKBP5, a co-chaperone of the glucocorticoid receptor in the pathogenesis and therapy of affective and anxiety disorders. Psychoneuroendocrinology, 2009 34: p. S186–S195. 10.1016/j.psyneuen.2009.05.021 19560279

