# Supplementary material for: Investigating the impact of early-life adversity on physiological, immune, and gene expression responses to acute stress: A pilot feasibility study
Source: PLoS One. 2020 Apr 3;15(4):e0221310. doi: 10.1371/journal.pone.0221310 (PMC7122782; doi:10.1371/journal.pone.0221310)
Supplement: S1 Table — Data presented as sMean (Standard Deviation). (DOCX) [file pone.0221310.s001.docx]

|  |  | **No Stress T1** | **No Stress T2** | **No Stress T3** | **No Stress T4** | **TSST T1** | **TSST T2** | **TSST T3** | **TSST T4** |
| --- | --- | --- | --- | --- | --- | --- | --- | --- | --- |
| **IL-1β** | ELA | 0.025 (0.04) | 0.063 (0.06) | 0.028 (0.04) | 0.045 (0.03) | 0.106 (0.04) | 0.116 (0.06) | 0.130 (0.09) | 0.113 (0.07) |
|  | Control | 0.047 (0.04) | 0.025 (0.04) | 0.047 (0.03) | 0.051 (0.04) | 0.110 (0.07) | 0.152 (0.06) | 0.149 (0.10) | 0.204 (0.09) |
|  | Full Sample | 0.037 (0.04) | 0.042 (0.05) | 0.038 (0.04) | 0.048 (0.04) | 0.108 (0.05) | 0.134 (0.06) | 0.140 (0.09) | 0.159 (0.09) |
| **IL-6** | ELA | 0.440 (0.30) | 0.446 (0.41) | 0.471 (0.41) | 0.524 (0.55) | 0.398 (0.12) | 1.863 (1.80) | 1.743 (1.16) | 1.682 (1.07) |
|  | Control | 0.307 (0.12) | 0.499 (0.23) | 0.917 (0.37) | 1.340 (0.63) | 0.778 (0.58) | 1.020 (0.66) | 1.571 (0.79) | 2.261 (0.92) |
|  | Full Sample | 0.368 (0.22) | 0.475 (0.31) | 0.714 (0.44) | 0.969 (0.71) | 0.588 (0.45) | 1.442 (1.37) | 1.657 (0.95) | 1.972 (1.00) |
| **IL-8** | ELA | 4.35 (1.40) | 3.88 (0.79) | 3.10 (1.59) | 3.27 (0.75) | 5.52 (1.14) | 6.41 (1.51) | 5.33 (1.92) | 5.42 (2.19) |
|  | Control | 4.82 (2.43) | 5.00 (3.85) | 5.95 (4.14) | 6.27 (2.88) | 7.65 (5.22) | 8.49 (6.05) | 8.77 (6.22) | 7.98 (3.62) |
|  | Full Sample | 4.60 (1.95) | 4.49 (2.83) | 4.65 (3.43) | 4.90 (2.61) | 6.59 (3.77) | 7.45 (4.34) | 7.05 (4.74) | 6.70 (3.15) |
| **TNFα** | ELA | 1.76 (0.49) | 1.83 (0.62) | 1.48 (0.39) | 1.51 (0.46) | 2.56 (0.71) | 2.81 (0.96) | 2.55 (0.96) | 2.69 (0.92) |
|  | Control | 1.61 (0.39) | 1.74 (0.45) | 1.75 (0.24) | 1.77 (0.14) | 2.71 (0.81) | 2.76 (0.76) | 2.85 (0.64) | 2.58 (0.61) |
|  | Full Sample | 1.68 (0.43) | 1.78 (0.51) | 1.63 (0.33) | 1.65 (0.33) | 2.63 (0.73) | 2.79 (0.83) | 2.70 (0.80) | 2.64 (0.75) |

**Supplementary Table 1**: Summary information for inflammatory cytokines (pg/mL) by time, session, and group status. Data presented as Mean (Standard Deviation).
