# Supplementary material for: Investigating the impact of early-life adversity on physiological, immune, and gene expression responses to acute stress: A pilot feasibility study
Source: PLoS One. 2020 Apr 3;15(4):e0221310. doi: 10.1371/journal.pone.0221310 (PMC7122782; doi:10.1371/journal.pone.0221310)
Supplement: S2 Table — Adjustment was performed across both primary outcomes (cortisol and MAP) and within each secondary outcome. Significant results are bolded. *Univariate analyses of raw cytokine AUCi were considered confirmatory following results of repeated measures models, and thus were not included in the count of total tests within raw cytokines secondary endpoint. (DOCX) [file pone.0221310.s002.docx]

| **Outcome** | **Effect Tested** | **Domain (Total Tests)** | **Original p-value** | **Bonferroni p-value** | **Passes Correction?** |
| --- | --- | --- | --- | --- | --- |
| Cortisol Repeated Measures | Session (Stress vs. No Stress) | Primary Endpoints (6) | **0.003** | 0.0083 | Yes |
| MAP Repeated Measures | Session (Stress vs. No Stress) |  | **0.003** | 0.0083 | Yes |
| MAP Repeated Measures | Status (Risk vs. Control; within TSST) |  | **<0.001** | 0.0083 | Yes |
| Cortisol ∆AUCi | Status (Risk vs. Control; across sessions) |  | 0.088 | 0.0083 | - |
| Cortisol Repeated Measures | Status (Risk vs. Control; within no stress) |  | 0.257 | 0.0083 | - |
| MAP Repeated Measures | Status (Risk vs. Control; within no stress) |  | 0.402 | 0.0083 | - |
|  |  |  |  |  |  |
| Repeated Measures NR3C1 | Stress vs. No Stress (whole sample) | Secondary Endpoint: Gene Expression (3) | **0.006** | 0.0167 | Yes |
| Repeated Measures NR3C1 | Stress vs. No Stress (control group only) |  | **0.013** | 0.0167 | Yes |
| Repeated Measures NR3C1 | Stress vs. No Stress (risk group only) |  | 0.406 | 0.0167 | - |
|  |  |  |  |  |  |
| Repeated Measures Principal Components | Session (Stress vs No Stress) | Secondary Endpoint: Cytokines PCA (7) | 0.831 | 0.0071 | - |
| AUCi 1^st^ Principal Component | Session (Stress vs No Stress) |  | 0.165 | 0.0071 | - |
| AUCi 2^nd^ Principal Component | Session (Stress vs No Stress) |  | **0.026** | 0.0071 | No |
| AUCi 1^st^ Principal Component | Risk vs Control (Stress only) |  | 0.667 | 0.0071 | - |
| AUCi 2^nd^ Principal Component | Risk vs Control (Stress only) |  | 0.548 | 0.0071 | - |
| AUCi 1^st^ Principal Component | Risk vs Control (No stress only) |  | 0.137 | 0.0071 | - |
| AUCi 2^nd^ Principal Component | Risk vs Control (No stress only) |  | 0.377 | 0.0071 | - |
|  |  |  |  |  |  |
| IL-6 Repeated Measures | Session (Stress vs No Stress) | Secondary Endpoint: Raw Cytokines (4*) | **0.044** | 0.0125 | No |
| IL-6 AUCi | Session (Stress vs No Stress) |  | **0.018** | 0.0125 | No |
| IL-1𝛽 Repeated Measures | Session (Stress vs No Stress) |  | 0.5 | 0.0125 | - |
| IL-1𝛽 AUCi | Session (Stress vs No Stress) |  | 0.257 | 0.0125 | - |
| IL-8 Repeated Measures | Session (Stress vs No Stress) |  | 0.47 | 0.0125 | - |
| IL-8 AUCi | Session (Stress vs No Stress) |  | 0.434 | 0.0125 | - |
| TNF-𝛼 Repeated Measures | Session (Stress vs No Stress) |  | 0.863 | 0.0125 | - |
| TNF-𝛼 AUCi | Session (Stress vs No Stress) |  | 0.537 | 0.0125 | - |

**Supplementary Table 2**: False discovery rate adjustment across study primary and secondary endpoints. Adjustment was performed across both primary outcomes (cortisol and MAP) and within each secondary outcome. Significant results are bolded. *Univariate analyses of raw cytokine AUCi were considered confirmatory following results of repeated measures models, and thus were not included in the count of total tests within raw cytokines secondary endpoint.
