# Supplementary material for: Investigating the impact of early-life adversity on physiological, immune, and gene expression responses to acute stress: A pilot feasibility study
Source: PLoS One. 2020 Apr 3;15(4):e0221310. doi: 10.1371/journal.pone.0221310 (PMC7122782; doi:10.1371/journal.pone.0221310)
Supplement: S3 Table — Original results shown at top. Results which vary in significance from main findings are bolded. (DOCX) [file pone.0221310.s003.docx]

|  | Age | SES | BMI | BAI | BDI | STAI | LESS | LEQ | PSS TSST | PSS No Stress |
| --- | --- | --- | --- | --- | --- | --- | --- | --- | --- | --- |
| **FULL SAMPLE** | **0.556** | **0.232** | **0.458** | **0.253** | **0.156** | **0.142** | **0.089** | **0.070** | **0.033** | **0.021** |
| Minus Participant 1 | 0.447 | 0.359 | 0.514 | 0.369 | 0.213 | 0.206 | 0.182 | 0.126 | 0.036 | 0.044 |
| Minus Participant 2 | 0.492 | 0.307 | 0.444 | 0.317 | 0.162 | 0.222 | 0.175 | 0.129 | 0.045 | 0.045 |
| Minus Participant 3 | 0.581 | 0.307 | 0.182 | 0.127 | 0.116 | 0.130 | 0.131 | 0.078 | **0.066** | 0.034 |
| Minus Participant 4 | 0.811 | 0.200 | 0.645 | 0.532 | 0.263 | 0.288 | 0.178 | 0.106 | **0.068** | 0.047 |
| Minus Participant 5 | 0.711 | 0.073 | 0.097 | 0.330 | 0.221 | 0.229 | 0.115 | 0.172 | **0.069** | 0.047 |
| Minus Participant 6 | 0.668 | 0.307 | 0.680 | 0.260 | 0.176 | 0.184 | 0.158 | 0.080 | 0.041 | 0.042 |
| Minus Participant 7 | 0.537 | 0.132 | 0.577 | 0.199 | 0.140 | 0.227 | **0.032** | **0.034** | **0.071** | 0.039 |
| Minus Participant 8 | 0.851 | 0.307 | 0.445 | 0.341 | 0.169 | 0.206 | 0.054 | 0.114 | **0.063** | 0.038 |
| Minus Participant 9 | 0.447 | 0.155 | 0.509 | 0.107 | 0.114 | **0.025** | 0.053 | **0.034** | 0.017 | 0.001 |
| Minus Participant 10 | 0.492 | 0.155 | 0.655 | 0.387 | 0.133 | 0.135 | 0.110 | 0.083 | 0.021 | 0.014 |
| Minus Participant 11 | 0.352 | 0.359 | 0.656 | 0.225 | 0.221 | 0.115 | 0.134 | 0.074 | 0.017 | 0.023 |
| Minus Participant 12 | 0.492 | 0.307 | 0.590 | 0.364 | 0.162 | 0.216 | 0.088 | 0.110 | **0.070** | 0.032 |

**Supplementary Table 3**: Leave one out sensitivity analyses of Sample Characteristics (p-value). Original results shown at top. Results which vary in significance from main findings are bolded.
