# Supplementary material for: Investigating the impact of early-life adversity on physiological, immune, and gene expression responses to acute stress: A pilot feasibility study
Source: PLoS One. 2020 Apr 3;15(4):e0221310. doi: 10.1371/journal.pone.0221310 (PMC7122782; doi:10.1371/journal.pone.0221310)
Supplement: S4 Table — Original results shown at top. Results which vary in significance from main findings are bolded. (DOCX) [file pone.0221310.s004.docx]

|  | Repeated Measures Cortisol  (Time x Session) | Repeated Measures MAP  (Time x Session) | Repeated Measures GR  (Time x Session) | Repeated Measures IL-6 (Time x Session) | Univariate IL-6 AUCi  (TSST vs. No Stress) |
| --- | --- | --- | --- | --- | --- |
| **FULL SAMPLE** | **p=0.003** | **p=0.003** | **p=0.006** | **p=0.044; eta=0.135** | **p=0.018; eta=0.240** |
| Minus Participant 1 | 0.018 | 0.006 | 0.020 | **p=0.126; eta=0.104** | p=0.030; eta=0.214 |
| Minus Participant 2 | 0.007 | 0.001 | 0.020 | **p=0.058; eta=0.138** | p=0.017; eta=0.264 |
| Minus Participant 3 | 0.011 | 0.002 | 0.011 | p=0.048; eta=0.147 | p=0.019; eta=0.258 |
| Minus Participant 4 | 0.007 | 0.005 | 0.006 | p=0.045; eta=0.150 | p=0.032; eta=0.221 |
| Minus Participant 5 | 0.004 | 0.013 | 0.008 | p=0.040; eta=0.151 | p=0.030; eta=0.224 |
| Minus Participant 6 | 0.004 | 0.004 | 0.013 | **p=0.075; eta=0.128** | p=0.021; eta=0.250 |
| Minus Participant 7 | 0.003 | 0.006 | 0.005 | p=0.032; eta=0.158 | p=0.021; eta=0.250 |
| Minus Participant 8 | 0.001 | 0.006 | 0.018 | **p=0.092; eta=0.121** | p=0.027; eta=0.233 |
| Minus Participant 9 | 0.018 | 0.003 | 0.004 | p=0.035; eta=0.159 | p=0.016; eta=0.268 |
| Minus Participant 10 | 0.001 | 0.003 | 0.012 | **p=0.088; eta=0.122** | p=0.024; eta=0.240 |
| Minus Participant 11 | 0.001 | 0.011 | 0.003 | p=0.037; eta=0.156 | p=0.039; eta=0.205 |
| Minus Participant 12 | 0.01 | p<0.001 | 0.003 | **p=0.059; eta=0.137** | p=0.016; eta=0.270 |

**Supplementary Table 4**: Leave one out sensitivity analyses of stress-induced changes in physiological, gene expression, and pro-inflammatory cytokines (p-value). Original results shown at top. Results which vary in significance from main findings are bolded.
