# Supplementary material for: Investigating the impact of early-life adversity on physiological, immune, and gene expression responses to acute stress: A pilot feasibility study
Source: PLoS One. 2020 Apr 3;15(4):e0221310. doi: 10.1371/journal.pone.0221310 (PMC7122782; doi:10.1371/journal.pone.0221310)
Supplement: S5 Table — Original results shown at top. Results which vary in significance from main findings are bolded. (DOCX) [file pone.0221310.s005.docx]

|  | Repeated Measures MAP  (Time x Status; TSST only) | Univariate Cortisol ΔAUCi  (ELA vs. Control) | Repeated Measures GR  (Time x Session; Control Only) | Repeated Measures GR  (Time x Session; ELA Only) |
| --- | --- | --- | --- | --- |
| **FULL SAMPLE** | **p<0.001** | **p=0.088** | **p=0.013; eta=0.36** | **p=0.406** |
| Minus Participant 1 | 0.001 | 0.154 |  | 0.426 |
| Minus Participant 2 | 0.001 | 0.139 | **p=0.071; eta=0.30** |  |
| Minus Participant 3 | p<0.001 | 0.162 | p=0.017; eta=0.38 |  |
| Minus Participant 4 | 0.002 | 0.178 |  | 0.454 |
| Minus Participant 5 | p<0.001 | 0.066 |  | 0.442 |
| Minus Participant 6 | 0.001 | 0.095 | p=0.039; eta=0.35 |  |
| Minus Participant 7 | 0.001 | **0.029** |  | 0.464 |
| Minus Participant 8 | p<0.001 | 0.088 | **p=0.052; eta=0.35** |  |
| Minus Participant 9 | p<0.001 | 0.18 |  | 0.354 |
| Minus Participant 10 | p<0.001 | 0.096 | p=0.028; eta=0.38 |  |
| Minus Participant 11 | 0.001 | 0.06 |  | 0.319 |
| Minus Participant 12 | 0.001 | 0.139 | p<0.001; eta=0.52 |  |

**Supplementary Table 5**: Leave one out sensitivity analyses of group differences in stress-induced changes in physiological, gene expression, and pro-inflammatory cytokines (p-value). Original results shown at top. Results which vary in significance from main findings are bolded.
